# Intracytoplasmic Crystalline Inclusions in the Hepatocytes of an Antelope

**DOI:** 10.4061/2010/373698

**Published:** 2010-12-27

**Authors:** Sanjeev Gumber, Eric Lombardini, Bruce Williams, Nobuko Wakamatsu

**Affiliations:** ^1^Department of Pathobiological Sciences, School of Veterinary Medicine, Louisiana State University, Skip Bertman Drive, Baton Rouge, LA 70803, USA; ^2^Armed Forces Institute of Pathology, Department of Defense, Washington, DC 20306-6000, USA

## Abstract

This case report describes intracytoplasmic crystalline inclusions in the hepatocytes of a 13-year-old female Thomson's gazelle. Histologically, multifocal to coalescing areas of many hepatocytes contained large cytoplasmic vacuoles filled with pale eosinophilic homogeneous material and rare fine basophilic granules. Von Kossa staining showed the presence of calcium within cytoplasm, mainly in the inclusions, of hepatocytes. Transmission electron microscopy, scanning electron microscopy, energy dispersive X-rays analyses, and infrared spectroscopy on the liver showed the hepatocellular material consistent with protein and carbohydrate with secondary accumulation of calcium and phosphorus. It was concluded that crystalline inclusions may have been derived due to failure of normal physiological hepatocellular clearance associated with a severe chronic disease. To the authors' knowledge this is the first reported case of hepatocellular crystalline inclusions in an antelope.

## 1. Introduction

Cytoplasmic crystalline inclusions and pathological calcification of liver have been described occasionally in humans. There are only a few reports of crystalline inclusions in hepatocytes of dogs [1], cattle [2, 3], chimpanzees [4], rats [5], rabbits [6], and Ohrid trout [7]. They have never been described in antelopes. The current report describes the histological and electron microscopic features of hepatocellular crystalline inclusions in a gazelle.

## 2. Case Description

**Figure 1:**
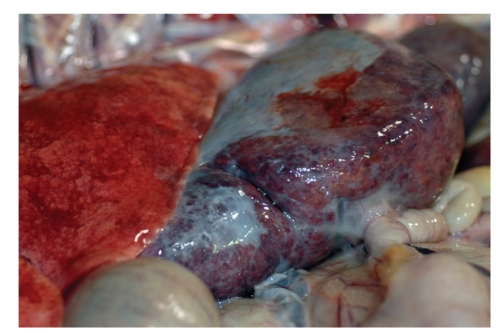
The liver has prominent rounded edges and a thick fibrous capsule.

**Figure 2:**
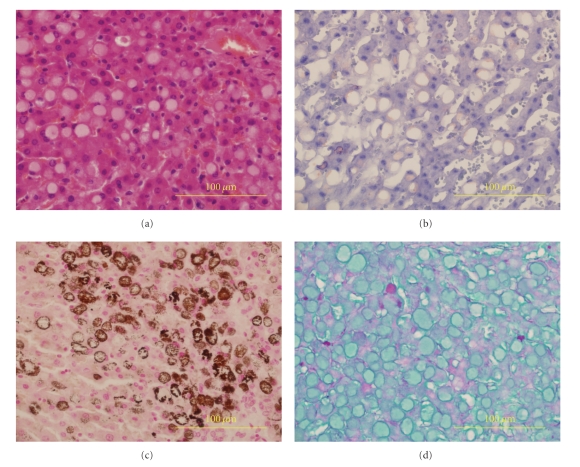
Liver: (a) large intrahepatic cytoplasmic vacuoles filled with pale eosinophilic homogeneous material. Hematoxylin and eosin stain; (b) minimal positive staining for fat in hepatic vacuoles. Oil red O stain; (c) dark brown to black staining indicating calcium (mineral) precipitate in hepatocytes. Von Kossa stain; (d) minimal positive staining for glycogen in hepatic vacuoles. PAS stain.

A 13-year-old female Thomson's gazelle (*Eudorcas thomsoni*) from a zoo was presented to Louisiana Animal Disease Diagnostic Laboratory, Louisiana State University (LSU) (Baton Rouge, LA, USA) for necropsy examination. The animal was found dead and had a history of severe parasitism. Postmortem examination revealed sparse body fat store in the gazelle. There was bilateral enlargement of thyroid glands (right: 6 × 3 × 2 cm; left: 2 × 2 × 1 cm), and the cut surfaces were pale tan with multifocal red areas. The liver was firm, rounded, and the cut surface was mottled tan and red (Figure 1). There was marked serous atrophy of fat in the bone marrow and epicardial fat. Histologically, multifocal to coalescing areas of many hepatocytes contained large cytoplasmic vacuoles filled with pale eosinophilic homogeneous material and rare fine basophilic granules with hematoxylin and eosin stain (Figure 2(a)). Periportal areas had moderately increased fibrous connective tissue with marked biliary hyperplasia, mild oval cell hyperplasia, and moderate infiltrates of predominant lymphocytes and plasma cells with occasional neutrophils. Multifocal small aggregates of neutrophils occasionally admixed with plasma cells, lymphocytes, and rare degenerative hepatocytes were present in the hepatic parenchyma. There were multifocal areas of hepatocellular nodular regeneration. The histological findings in liver were diagnosed as chronic multifocal to coalescing severe hepatocellular vacuolation with unknown material and mineral, and multifocal moderate lymphoplasmacytic cholangiohepatitis, bridging portal fibrosis and biliary hyperplasia. There was no evidence of hepatocellular necrosis. The histopathological findings in liver were interpreted as severe chronic hepatic disease. The majority of intracytoplasmic inclusions failed to stain for fat with oil red O (Figure 2(b)), glycogen with periodic acid-Schiff stain (with and without diastase digestion) (Figure 2(d)), and acid mucopolysaccharides. The hepatocytes stained strongly with von Kossa staining consistent with calcium (mineral), mainly in the intracytoplasmic inclusions (Figure 2(c)). Additional stains including Hall's for bile pigment, fibrinogen, phosphotungstic acid hematoxylin for fibrin, Prussian blue for iron, auramine-rhodamine for acid fast organisms, Mayer's mucicarmine for polysaccharide, alcian blue (pH 2.5) for mucin, and congo red for amyloid failed to stain the pale eosinophilic cytoplasmic inclusions. Additionally, immunohistochemical staining for fibrinogen, kappa, and lambda light chains yielded negative results. An undetermined storage disease was considered as a differential in the tissue sections of liver. The enlarged thyroid glands were diagnosed as thyroid hyperplasia. No other significant microscopic findings were seen in this animal. Fecal floatation revealed moderate mixed parasitic burden including *Trichostrongylus* spp., *Haemonchus* spp., and *Nematodirus* spp. parasites eggs. 

**Figure 3:**
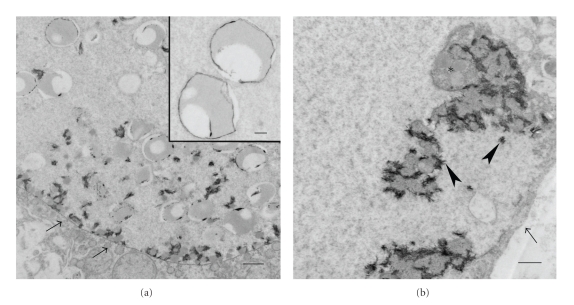
Liver: (a) cytoplasmic crystalline inclusions within a hepatocellular vacuole (arrows). Bar: 2 *μ*m; Inset: higher magnification of inclusions. Bar: 500 nm. (b) the hepatocellular vacuole (arrow) containing intermediate electron-dense lipid (∗) with highly electron-dense mineral (arrowheads). Bar: 1 *μ*m. Uranyl acetate-lead citrate stain. Transmission electron micrograph.

For transmission electron microscopic examination, 1-mm^3^ fragments of liver were fixed in 3% glutaraldehyde, postfixed in 1% osmium tetroxide, dehydrated in ethanol, and embedded in liquid epoxy resin. The ultrathin sections (70–90 nm) were stained with lead citrate and uranyl acetate and examined with an electron microscope (JEOL JEM-1011). Ultrastructurally, nonmembrane bound, irregular shaped crystalline inclusions were seen in the cytoplasmic vacuoles of hepatocytes (Figure 3(a)). Lipid with/without mineral deposits was noted within the vacuoles (Figure 3(b)). Scanning electron microscopy and energy dispersive X-rays analyses (SEM/EDXA) on the liver provided evidence of accumulations of calcium, oxygen, phosphorus, and smaller amounts of magnesium and sodium. Infrared spectroscopy showed the hepatocellular material consistent with protein and carbohydrate.

## 3. Discussion

Hepatocellular cytoplasmic crystalline inclusions have been described in cattle with mucosal disease [2], 8-month-old steer with shipping fever [3], dogs and rats with *Escherichia coli* endotoxin [5], and liver of normal dogs [8] and rabbits [6]. The presented case was an 11-year-old gazelle; therefore, it appears that there is unknown relationship between hepatocellular inclusions and age of the animal. Crystalline inclusions have been observed in the hepatocytes of normal human liver, but more frequently in Gilbert's syndrome [9]. Gilbert's syndrome is a congenital hyperbilirubinemia seen in humans, inherited as an autosomal dominant trait [10]. It was suggested that disruption of mitochondria liberates the inclusions into the cytoplasm or that crystals represent some form of hepatic degeneration in humans [9] or crystallized form of viruses [11]. Nonmembrane bound hepatocellular intracytoplasmic crystalline inclusions have never been reported in an antelope. Crystalline inclusions in the mitochondria of dog's liver have been documented in animals kept on low-protein diet [12]. However, the crystalline inclusions in the present study were extramitochondrial, and their pattern was different from the structure of intramitochondrial inclusions described in dogs. SEM/EDXA analyses on the liver of gazelle provided evidence of accumulations of calcium, oxygen, phosphorus, and smaller amounts of magnesium and sodium. Infrared spectroscopy showed the hepatocellular material consistent with protein and carbohydrate. There was no evidence of bacterial or viral infection microscopically and ultrastructurally. Bacteriological culture on liver revealed no growth. Initially, storage disease was considered as a possible differential after examining the tissue sections of liver stained with hematoxylin and eosin. Storage diseases can be either inherent/spontaneous or acquired/toxin induced (such as due to ingestion of toxic plants). There was no clinical history of toxin exposure in the zoo, but it cannot be completely excluded. 

The possible cause of severe emaciation of this gazelle was considered due to severe parasitism and concurrent thyroid hyperplasia. At the time of necropsy, there was a moderate parasitic burden in this animal based on fecal examination. However, with the history of recent deworming, the number of worms and ova remaining could be indicative of a severe infestation earlier. Hepatocellular calcification has been reported following a wide range of injuries such as inflammatory state, benign, and malignant neoplasm [13]. In the majority of the cases, calcification was consequence of massive hepatocellular necrosis or apoptosis [14]. However, there was no evidence of necrosis and/or apoptosis in the present case. The hepatic lesions in this gazelle were most likely due to a combination of thyroid hyperplasia and severe/chronic parasitism leading to hypoproteinemia. This finding is in partial agreement with a previous study in which dogs fed on low-protein diet developed intramitochondrial crystalline inclusions [12]. The cytoplasmic hepatocellular crystalline inclusions in the current report could be due to altered lipoprotein synthesis through disturbed oxidative metabolism in addition to disorganization of the membranous compartments in the cytoplasm. The other possible mechanism is that crystalline inclusions may have been derived due to failure of normal physiological hepatocellular clearance associated with a severe chronic disease which was confirmed histologically in the present case. It is also intriguing to speculate that increased protein production and increased permeability of nuclear membrane caused cytoplasmic crystallization of the protein. Moreover, the coexistence of calcium and phosphorus on SEM/EDXA analyses in the liver is suggestive of precipitation of calcium and phosphorus within the cytoplasm of hepatocytes. Whether the crystalline inclusions are de novo or degenerative products of the preexisting material in the cells remains to be elucidated. This case report is the first to describe cytoplasmic hepatocellular crystalline inclusions in an antelope.
